# Analytical Analysis of Motion Separability

**DOI:** 10.1155/2013/878417

**Published:** 2013-11-21

**Authors:** Marjan Hadian Jazi, Alireza Bab-Hadiashar, Reza Hoseinnezhad

**Affiliations:** School of Aerospace, Mechanical and Manufacturing Engineering, RMIT University, Corner of Plenty Road and McKimmies Road, Bundoora, Victoria 3083, Australia

## Abstract

Motion segmentation is an important task in computer vision and several practical approaches have already been developed. A common approach to motion segmentation is to use the optical flow and formulate the segmentation problem using a linear approximation of the brightness constancy constraints. Although there are numerous solutions to solve this problem and their accuracies and reliabilities have been studied, the exact definition of the segmentation problem, its theoretical feasibility and the conditions for successful motion segmentation are yet to be derived. This paper presents a simplified theoretical framework for the prediction of feasibility, of segmentation of a two-dimensional linear equation system. A statistical definition of a separable motion (structure) is presented and a relatively straightforward criterion for predicting the separability of two different motions in this framework is derived. The applicability of the proposed criterion for prediction of the existence of multiple motions in practice is examined using both synthetic and real image sequences. The prescribed separability criterion is useful in designing computer vision applications as it is solely based on the amount of relative motion and the scale of measurement noise.

## 1. Introduction

Computer vision problems in general involve manipulation of complicated manifolds. However, due to the mathematical and computational complexities of finding solutions in those spaces, a large group of problems are solved via projections leading to approximate solutions found by solving systems of linear equations. For example, solutions of important computer vision problems such as optical flow [1], fundamental matrix [2], and parametric image segmentation of man-made objects [3] are commonly found by solving systems of linear equations. After decades of research in these areas, a rich collection of methods to both robustly and efficiently solve those problems is currently available [4, 5].

Substantial efforts are also devoted to solve the motion segmentation problem [6]. A major shortcoming of those solutions stems from the fact that structures in visual data are not precisely defined. The segmentation methods are typically considered successful when those methods are able to partition data in a way that by visual *inspection*, segments are deemed to be part of an identifiable object. As such, there is no theory as yet to predict which two motions are separable for a given set of data or what would be the minimum relative velocity that would constitute another motion. This question has important engineering applications particularly for designing devices that use visual measurements of speed, such as visual traffic surveillance systems, as a source of information.

Generally speaking with any probability distribution with infinite tails such as commonly used Gaussian, there will always be a finite probability of misclassified data no matter what the separation. The “magic bullet” of a clean threshold where “it makes sense to declare two structures as separate and assign points, and on the other side of the threshold you can't distinguish” is incompatible with this model. The concept of separability itself is also not well defined and one can identify at least two different (but related) notions as outlined here.

1. Detectability: existence of two structures (motions) in a given data is detectable, but the detected structures are not necessarily distinguishable.
2. Separability: structures (motions) can be distinguished from each other and the segmentation of data between different structures can be performed up to a desired (given) level of reliability.

In engineering applications, the latter notion of separability is of significant interest. The characteristics of the structures are important and those characteristics cannot be measured unless the structures are well defined. The misclassification error of separating two models in general will be governed by fitting errors of the two putative models as well as the overlap of the distributions. Although the deciding line is always going to be somewhat arbitrary and dependent on what the engineering problem will tolerate, this paper makes an important contribution by quantifying the probability of misclassification. Our theoretical derivations also show that the separability problem is very complicated and development of an elegant solution that could predict separability in all cases does not appear to be straightforward. We have however been able to show how the Gaussian error of data relates to error of pairwise estimates of model parameters and how to quantify the exact overlap of their distributions. This at least enables us to identify the crossover point which is a useful guide for defining a structure and ultimately designing reliable equipment. We explain the use of crossover point in section 3-B, after we derive the above relationship.

Optical flow calculation has been one of the most studied problems of computer vision and its calculation [4, 7–10] and segmentation [11, 12] and the derivation of its confidence measures (error quantification) [13, 14] have been refined over several decades. However, the limits of using optical flow knowledge as a primary source of physical perception are yet to be established. More importantly, where there are at least two motions in a local area, it is currently not possible to predict how much difference between those motions is required for those motions to be separable.

**Figure 1:**
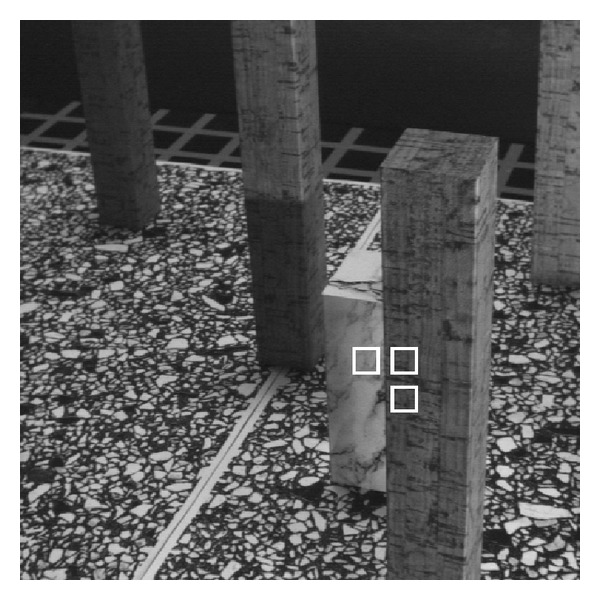
A frame of the Marbled-Block image sequence where three local areas, two on one column having the same motion and one on a different column with distinctly different motions, are highlighted by white rectangles. The main question here is to predict the least amount of relative motion between the two columns that would make those definitely separable by their motions.

For instance, as shown in Figure 1, one would need to know how much relative motion between different objects in this image would be required to reconstruct the scene geometry using motion information. In this figure, we have highlighted three local areas where two are on one column (having very similar motion) and the other on a different column. The question here is to ascertain how much difference in motion between these objects would be required to distinguish their motions in the given situation.

The issue of finding the limits of detection for near discontinuities in visual data was first discussed in [15]. The work focused on range data segmentation and the separability criteria were only derived for special cases including parallel or crease structures. The above work was later extended to study the effect of consistency [16] and finite sample bias [17] of commonly used estimators on the separability of close structures. However, those works were also limited to the special case of parallel discontinuities only. Although the optical flow segmentation is a dual problem of the range segmentation discussed in [15], the underlying structures in motion segmentation are not limited to parallel structures and our aim here is to develop general analysis that is independent of the segmentation strategy.

To properly address the separability issue, we first need to have a precise definition of a *structure*. Having defined a structure, we then need to establish the general conditions for the separability of those structures for cases where nearby structures exist.

We focus on answering the above question for the two most similar motions in an image which their motion estimations are modeled as an instance of the optical flow problem. In essence, we aim to find a general condition for segmentation of motion when the two motions are modeled by optical flow constraints and formulated as a solution of a linear system of equations. The overall scope is therefore much broader and includes the ubiquitous problem of confirming the existence of multiple close solutions in a system of linear 2D equations in the presence of noise and outliers.

The rest of this paper is organized as follows. The motion separability problem is formulated in Section 2. A solution for the prediction problem is presented in Section 3 followed by the results of usability experiments using both synthetic and real data presented in Section 4.  Section 5 concludes the paper.

## 2. Problem Formulation: Motion Separability

 The current trend in optical flow estimation is to use nonparametric representations. Those methods (e.g., [4, 7–10]) often use either variational or discrete optimization methods to find solutions that show a degree of smoothness across motion boundaries by imposing certain regularization terms. These approaches involve tuning a large number of parameters that their optimum values depend on the structure of a scene. The analysis of a variety of optical flow estimation techniques has however shown that “only a small number of key choices produce statistically significant improvements” in the overall accuracy of those methods [9]. More importantly, the above analysis has also shown that applying a median filter to intermediate flow values produces the most significant improvement. This implies that the appropriate separation of different motions is a key ingredient of the estimation process.

Since our aim here is to quantify the separability of two motions, we need to disentangle the optical flow estimation from the scene dependant implications of smoothness imposed implicitly by the nonparametric methods. To achieve this aim, we start the motion separability analysis by modeling the optical flow problem in its classical form presented in [18] without imposing an arbitrary smoothness across the motion boundaries. The local optical flow, without smoothness imposition, is modeled as a solution of a linear system:

(1) $$\begin{array}{c} A\nu =b+\epsilon \end{array}$$

in which *A* is a matrix with two columns, each containing the spatial derivatives (*I*_*x*_ and *I*_*y*_) in directions of the velocity *ν* components (*u*, *v*), *b* is a vector of associated temporal derivatives, and *ϵ* represents the noise [18]. In this set up, if there are two motions, the above system of equations must be separable into two systems of linear equations and the separability would be a function of the difference of those motions. Intuitively, if the difference is very small, compared to the accuracy of estimation, the variations would be similar to noise and they would not be separable. Otherwise, it should be possible to separate those sets of equations.

Our aim here is to find the sufficient condition for the separability of two motions and as such, we use the most parsimonious motion model, constant flow, as it represents the least accurate (most inseparable) scenario. Models with more parameters (e.g., affine) are expected to produce better estimates of motions and therefore their separability condition is covered by the above model separability condition.

**Figure 2:**
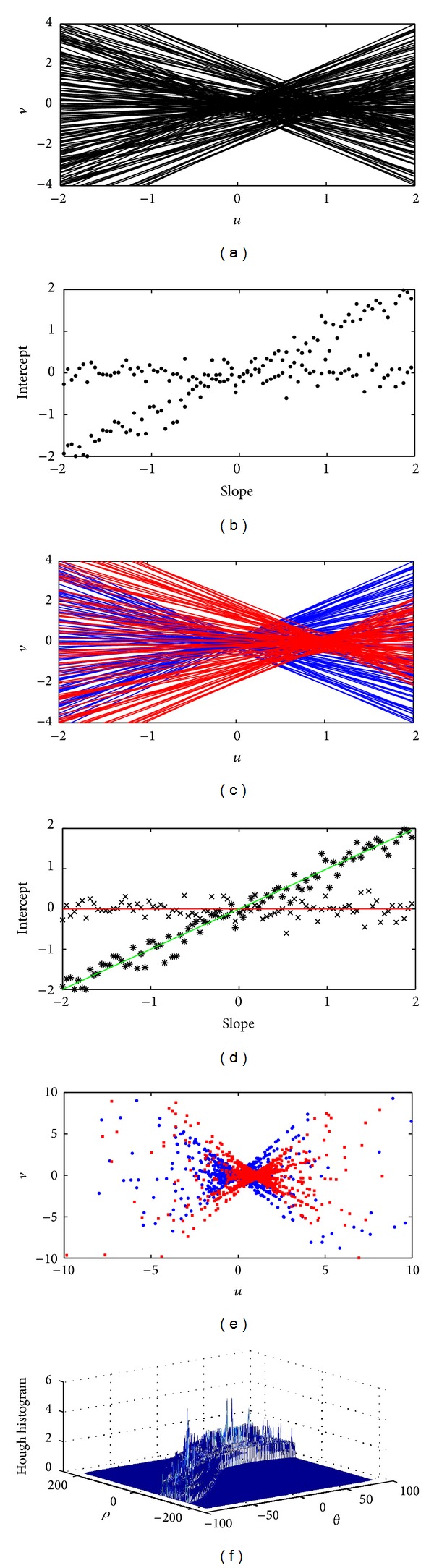
(a) Geometric representation of a 2D system of linear equations with two solutions. (b) The dual representation of the same system of linear equations in parameter space. In absence of an exact definition for a data structure, the underlying structures of these two figures can be visually interpreted in many different ways. (c) and (d) An interpretation of the above figures visualized based on linear models and added Gaussian noise. (e) Intersections of all the lines shown in (a) and (c) where blue and red markers represent the intersections of blue and red lines with themselves. (f) Histogram of putative solutions in the Hough space.

For a local area containing multiple motions, the system of (1) would contain a number of coherent subsystems which would appear as different clusters of cointersecting lines in the (*u*, *v*) plane (see Figure 2(a)). To formulate the separability problem, without loss of generality, we can assume that there are only two motions in the area. The extent of the area is not fixed and the ones considered are the closest motions and therefore the hardest to distinguish from each other. If there are more motions, they are by definition further apart and would not affect the outcome. In this setting, the effect of other possible motions would be similar to gross outliers and are not expected to have a major impact on the separability issue as optical flow calculation methods are typically robust to the influences of outliers [7].

The motion separability problem, in its abstract form, is now represented by the problem of predicting the separability of two clusters of cointersecting lines based on two factors: the “distance” (the relative motion) between those clusters and the extent of the spread of constraint lines in each cluster (measurement noise). In its dual space, as shown in Figure 2(b), this problem is equivalent to the separability of two linear structures in a cloud of 2D points. To the best of our knowledge, currently there is no theory to predict the separability of those line clusters generally.

A common approach to tackle the above problem, in its dual form, is to use the Hough transform [19, 20]. In this approach, the Hough domain is first *uniformly* quantized into number of cells and each cell maintains a count of passing lines. The center of the cell with the largest number of counts is an estimate of the underlying structure parameters. To detect multiple structures, a threshold is specified and cells with counts exceeding a threshold are considered as putative structures. This approach has a major drawback stemming from the fact that the probability density function of the structure parameters has to be estimated using a discrete histogram as shown in Figure 2(f). As the figure shows, the interpretation of the histogram for the detection of different motions would heavily depend on the values of manually set thresholds.

To address this issue, Dahyot [20] has proposed a form of statistical kernel modeling for the Hough transform to estimate a continuous histogram. The proposed approach needs two pieces of information: the shape of a suitable kernel and the appropriate bandwidth. The overall approach is able to accurately estimate the parameters by locating the peaks of the histograms in cases where the structures are distinct. However, the choices of kernel shape and bandwidth have significant impact on the spread of the final pdf which is the key information for the separability analysis. As such, kernel density estimation appears not to be an appropriate tool for separability prediction.

To overcome the above problems, we propose to use a discrete part of the parameter space spanned by the exhaustive sampling of possible intersections as partly shown in Figure 2(e). To devise the separability condition for the optic flow problem, we derive the probability distribution of those samples as well as providing a precise definition for a structure (motion) in the space of those samples. The combination of these two pieces of information enables us to quantify the least amount of relative motion that would be separable for a given set of data. We will then show that those derivations are useful for predicting separability in practical situations.

## 3. Separability Prediction

An important aspect of replacing clusters of lines with all their intersection points (using existing parameter space discretization) is that the process changes the nature of the problem from finding the solution of an overdetermined system of linear equations (with multiple solutions) to a much simpler position estimation problem with multiple clusters. In the position estimation problem, the prediction of separability is fairly straight forward and the only information it requires is the definition of a cluster and the distributions of data points (line intersections).

**Figure 3:**
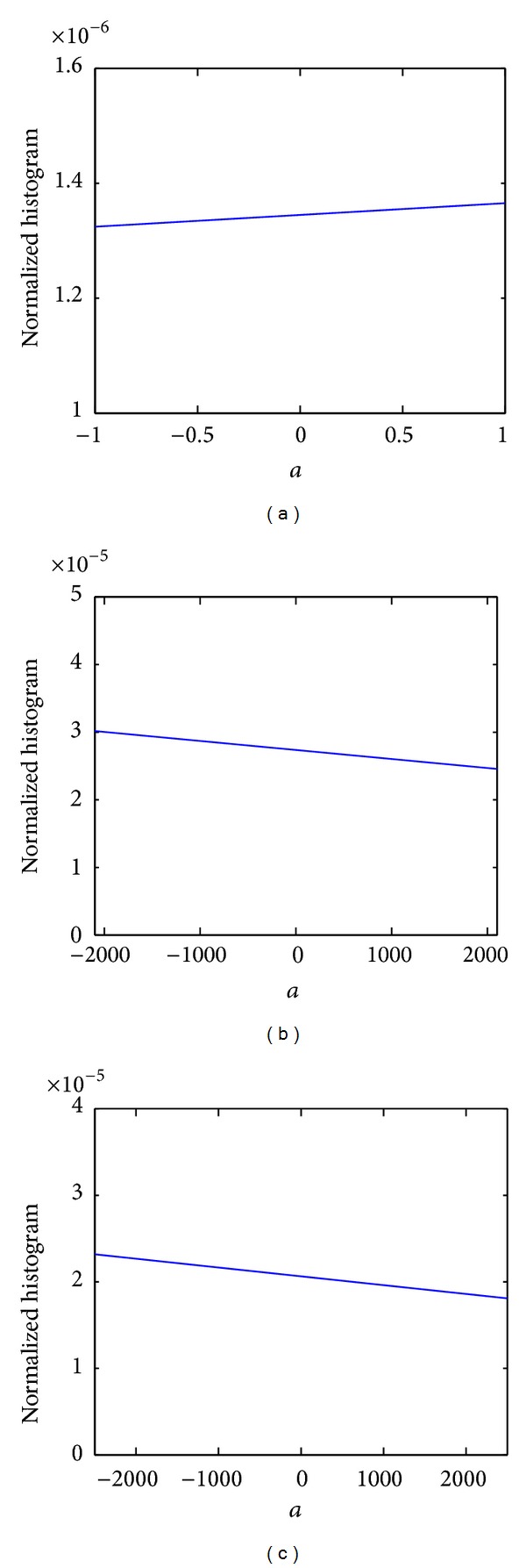
Normalized histogram of *a* = *I*_*x*_/*I*_*y*_ for a number of image sequences commonly used in optical flow evaluations including; (a) RubberWhale (Middlebury), (b) Grove2 (Middlebury), (c) Marbled Block [22].

Our practical intuition for solving this problem comes from our experiments with a variety of gray scale and color images commonly used for optical flow evaluations [21, 22]. We observed that in all of these images, the ratio of spatial derivatives of image intensity function (ratios of *I*_*x*_ and *I*_*y*_ for different pixels), to a large degree, is uniformly distributed over a fairly broad range of values. To provide some evidence, examples of the distribution of the ratios of the spatial derivatives of different image sequences in the Middlebury dataset are shown in Figure 3.

Although there might be small patches in some specific images in which the texture has a dominant direction (e.g. parallel strips), overall the commonly experienced textures have derivatives in all directions and very little can be generalized about the ratio of those derivatives. In fact, from an information point of view, assuming a uniform distribution is the least *restrictive* assumption one can make about the characteristics of those textures.

To establish the separability condition for linear structures (e.g., motions modeled by optical flow), we first replace all lines with their pairwise intersection points and then derive the probability distribution of those points by assuming that the ratio of spatial derivatives is uniformly distributed. The system of (1) is then replaced by a cluster of $N=\left(\begin{array}{c} n\\ 2 \end{array}\right)$ points and the problem of finding the best solution to the above system based on a measure of goodness (e.g., least squares) is transformed to an instance of a well-known position estimation problem.

### 3.1. Error Distribution

To derive the distribution of the intersections points (for an equation system with one solution), we denote the true velocity by $\hat{v}$, the distance between a point and the true velocity by *r*, and the probability density function by *f*(*r*). This distance represents the error in the direction that the motion separability is examined in. If one of the components of the two motions is separable, then those motions will also be separable. In our derivations, we perform the calculations for an arbitrary direction and the rationale is that the separability is always tested in a particular direction. For instance, if the optical flow calculation is used to monitor highway traffic, the separability of horizontal velocities of different vehicles is of interest to the system designer. The differences of velocities in other directions (e.g., vertical in this case) are of no significance. The design parameter for this system is the scale of measurement noise for one vehicle in that particular direction and it depends on many factors including the quality of cameras, vehicles textures, and visibility conditions. The above design parameter (scale of measurement noise) captures the overall effect of those factors.

It is important to note that for the sake of simplicity, we model the flow calculation and its errors in the classical regression framework rather than the more accurate geometric modeling framework [23, 24]. This simplification is justified because the regression model for optical flow calculations is almost as accurate as the geometric model as long as the optical flow constraints are not parallel (or near parallel) to the vertical axis [5]. Although values of the ratio of spatial partial derivatives of image intensity function (ratios of *I*_*x*_ and *I*_*y*_ for different pixels) are between plus and minus infinity, values between plus and minus one span half the velocity space. In this half space, the lines are away from the vertical direction and the classical regression modeling is accurate. We conduct our calculations for the above interval and later extend those to the whole velocity space.

To develop a solution to the above separability problem, we first introduce and prove a proposition regarding the distribution of pairwise samples. We then use the result to define structure and devise a separability criterion. Suppose that (*a*_1_, *b*_1_),…,(*a*_*n*_, *b*_*n*_) are independent identically distributed random observations drawn from the model

(2) $$\begin{array}{c} v=a_{i}u+b_{i}+e_{i}, \end{array}$$

where

- *e* is normally distributed with zero mean and known variance *σ* ^2^ (although noise of different optical flow constraints is likely to be correlated, this assumption simplifies the modeling (of an otherwise intractable problem) while its computational bias is shown to be relatively small [9, 25]. Our definition of a structure, provided later, is inherently robust to influence of large perturbations and would not include samples that might have been generated by degenerate constraints. Our experiments with both synthetic and real images have also shown that this assumption does not generate significant bias in the final results);
- *a* is uniformly distributed.

**Proposition 1**

Under assumptions (i) and (ii), the estimation error *r* (difference between the estimated value *v* and the real value $\hat{v}$) has the following distribution:

(3) $$\begin{array}{c} f_{r}\left(r\right)=\frac{\sigma }{2\sqrt{2\pi }r^{2}}\left(1-e^{-2r^{2}/\sigma^{2}}\right). \end{array}$$

**Proof**

Using (2), the coordinates of the intersection of lines *i* and *j* are

(4) $$\begin{array}{c} v_{k}=b_{i}+e_{i}+a_{i}\frac{b_{j}-b_{i}}{a_{i}-a_{j}}+a_{i}\frac{e_{j}-e_{i}}{a_{i}-a_{j}}, \end{array}$$

and the estimation error for the *k*th point (which is the intersection of *i* and *j* lines) can be written as

(5) $$\begin{array}{c} r_{k}=v_{k}-\hat{v}=e_{j}\frac{1}{1-\left(a_{j}/a_{i}\right)}+e_{i}\frac{1}{1-\left(a_{j}/a_{i}\right)}. \end{array}$$

It is important to note that values of *a*_*i*_ denote the slope of linear equations and values between ±1 represent constraints that cover half the space between *v* = *u* and *v* = −*u*. Since the estimation error is not a function of the chosen coordinate system, the distribution of error should also be the same in the other half of space. We use this fact and simplify the derivations by first finding the distribution where *a* ~ *𝒰*(−1,1). The estimation error distribution for other values of *a* will be identical.

To derive the distribution of the *r*, we denote

(6) αk=11−(aj/ai),βk=1−aiaj

and first find the distribution of *β*. The distribution of *α* is then calculated as the inverse of *β*.

If the probability density function of a random variable *X* is denoted by *f*_*X*_(*x*), the probability density function of *Y* = *g*(*X*) is as follows [26]:

(7) $$\begin{array}{c} f_{Y}\left(y\right)=\left|\frac{d}{dy}\left(g^{-1}\left(y\right)\right)\right|f_{X}\left(g^{-1}\left(y\right)\right). \end{array}$$

Using the above equation, the relationship between distributions of *α* and *β* can now be written as

(8) $$\begin{array}{c} f_{\alpha }\left(\alpha \right)=\frac{1}{\alpha^{2}}f_{\beta }\left(\frac{1}{\alpha }\right). \end{array}$$

And since *a* is assumed to have uniform distribution *a* ~ *𝒰*(−1,1) (i.e., half the space), the probability density function of *β* can be calculated as

(9) $$\begin{array}{c} f_{\beta }\left(\beta \right)=\{\begin{array}{cc} \frac{1}{4} & \text{if}\;0\leq \beta \leq 2,\\ \frac{1}{4{\left(1-\beta \right)}^{2}} & \text{If}\{\begin{array}{c} \beta <0\\ \beta >2. \end{array} \end{array} \end{array}$$

Substituting in (8), we have

(10) $$\begin{array}{c} f_{\alpha }\left(\alpha \right)=\{\begin{array}{cc} \frac{1}{4\alpha^{2}} & \text{if}\;\alpha \geq \frac{1}{2},\\ \frac{1}{4{\left(\alpha -1\right)}^{2}} & \text{if}\;\alpha <\frac{1}{2}. \end{array} \end{array}$$

To calculate the pdf of different terms in (5), we note that the probability density function of the product of two independent random variables *X* and *Y* (*Z* = *XY*) is as follows [26]:

(11) $$\begin{array}{c} f_{Z}\left(z\right)=\int_{-\infty }^{\infty }\frac{1}{\left|x\right|}f_{X}\left(x\right)f_{Y}\left(\frac{z}{x}\right)dx. \end{array}$$

Rewriting (5), we have

(12) $$\begin{array}{c} r_{k}=e_{i}\alpha_{i}+e_{j}\left(1-\alpha_{i}\right), \end{array}$$

and the probability density function of *ρ* = *eα* can be written by using (11), as

(13) fρ(ρ)=∫−∞∞1|α|12πσ2    ×e−ρ2/2α2σ2fα(α)dα.

Substituting (10) in (13), we have

(14) fρ(ρ)=∫−∞1/21|α|14(α−1)212πσ2e−ρ2/2α2σ2  dα   +∫1/2∞1α14(α)212πσ2e−ρ2/2α2σ2  dα.

The integrand of the second integral is continuous in its domain and the integration result is as follows:

(15) ∫1/2∞1α14(α)212πσ2e−ρ2/2α2σ2dα =σ42πρ2(1−e−2ρ2/σ2).

The first integral however has a discontinuity at zero and is generally intractable. Nonetheless, we have found a neat approximation to solve this integration problem. To outline our solution, we first denote the integrand by

(16) $$\begin{array}{c} g\left(\alpha ,\rho \right)=\frac{1}{\left|\alpha \right|}\frac{1}{4{\left(\alpha -1\right)}^{2}}\frac{1}{\sqrt{2\pi \sigma^{2}}}e^{-\rho^{2}/2\alpha^{2}\sigma^{2}} \end{array}$$

and note that the Dirac delta function can be written as

(17) $$\begin{array}{c} \delta \left(x\right)=\underset{\epsilon \to 0^{+}}{\lim}\frac{1}{2\sqrt{\pi \epsilon }}e^{-x^{2}/4\epsilon }. \end{array}$$

Combining (17) and (16), we have

(18) $$\begin{array}{c} \underset{\alpha \to 0}{\lim}g\left(\alpha ,\rho \right)=\frac{1}{\sqrt{2}\sigma }\delta \left(\frac{\sqrt{2}\rho }{\sigma }\right). \end{array}$$

**Figure 4:**
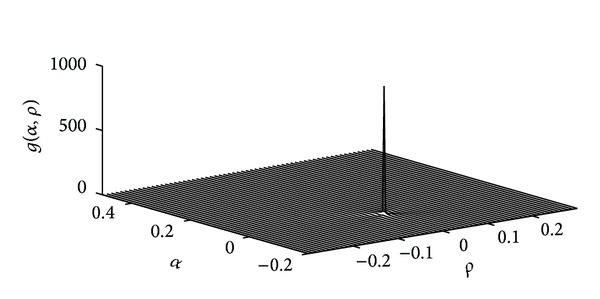
The shape of the integrand of the first integral in (14) for *σ* ^2^ = 1.

We also note that, as it is shown in Figure 4, *g*(*α*, *ρ*) is almost zero everywhere except around the origin. Inspired by this and (18), we approximate this two-dimensional discontinuity by a multiplication of two delta functions in every dimension. We later show that this is a very accurate approximation in terms of the total sum of probabilities. Consequently, we assume

(19) $$\begin{array}{c} g\left(\alpha ,\rho \right)\approx \{\begin{array}{cc} \frac{1}{\sqrt{2}\sigma }\delta \left(\frac{\sqrt{2}\rho }{\sigma }\right)\delta \left(\alpha \right) & \text{if}\;\alpha \to 0,\rho \to 0,\\ 0 & \text{O}.\text{W}. \end{array} \end{array}$$

and the result of the first integral in (14) can now be calculated as

(20) ∫−∞1/2g(α,ρ)dα=       ∫−∞1/212σδ(2ρσ)       δ(α)dα=12σδ(2ρσ).

To show that the above approximation is accurate in terms of the total probabilities, we first note that the sum of probabilities associated with the second integral in (14), which was calculated exactly, is 1/2 since

(21) $$\begin{array}{c} \int_{-\infty }^{\infty }\frac{\sigma }{4\sqrt{2\pi }\rho^{2}}\left(1-e^{-2\rho^{2}/\sigma^{2}}\right)d\rho =\frac{1}{2}. \end{array}$$

Therefore, the sum of probabilities associated with the first integral in (14) must also be 1/2. Importantly, the above approximation satisfies this requirement as shown below:

(22) $$\begin{array}{c} \int_{-\infty }^{\infty }\frac{1}{\sqrt{2}\sigma }\delta \left(\frac{\sqrt{2}\rho }{\sigma }\right)d\rho =\frac{1}{2}. \end{array}$$

Having calculated both integrals of (14), the probability density function of *ρ* = *eα* can be written as

(23) $$\begin{array}{c} f_{\rho }\left(\rho \right)=\frac{1}{\sqrt{2}\sigma }\delta \left(\frac{\sqrt{2}\rho }{\sigma }\right)+\frac{\sigma }{4\sqrt{2\pi }\rho^{2}}\left(1-e^{-2\rho^{2}/\sigma^{2}}\right). \end{array}$$

To find the distribution of all residuals (12), using the law of total probability, we write

(24) $$\begin{array}{c} f_{\rho }\left(\rho \right)=\frac{1}{2}f_{\rho }\left(\rho \;|\;\alpha >\frac{1}{2}\right)+\frac{1}{2}f_{\rho }\left(\rho \;|\;\alpha >\frac{1}{2}\right), \end{array}$$

and by defining *ρ*_1_ and *ρ*_2_ to be

(25) ρ={ρ1if  α>12,ρ2if  α<12,

we can rewrite (24) as

(26) $$\begin{array}{c} f_{\rho }\left(\rho \right)=\frac{1}{2}f_{\rho_{1}}\left(\rho_{1}\right)+\frac{1}{2}f_{\rho_{2}}\left(\rho_{2}\right) \end{array}$$

and comparing this with (14), we have

(27) fρ1(ρ1)=σ22πρ12(1−e−(2ρ12/σ2))fρ2(ρ2)=22σδ(2ρ2σ).

To derive the pdf of estimation errors *r* given by (12), using the law of total probability, we can write

(28) fr(r)=fr(eiαi+ej(1−αi))=12fr(eiαi+ej(1−αi) ∣ αi>12) +12fr(eiαi+ej(1−αi) ∣ αi>12).

Equation (10) shows that *α* and (1 − *α*) have the same distribution and (1 − *α*) is greater than 1/2, when *α* < 1/2. Using definition (25), the above equation can then be rewritten as

(29) fr(r)=12fr(ρ1+ρ2)+12fr(ρ2+ρ1)=f(ρ1+ρ2).

We note that since *ρ* values are independent and identically distributed (iid) random variables, *ρ*_1_ and *ρ*_2_ can also be considered iid random variables. Therefore, the distribution of the *r* is the convolution of the two distributions given by (27):

(30) fr(r)=fρ1∗fρ2   =22σδ(2rσ)∗σ22πr2(1−e−2r2/σ2),

(31) fr(r)=σ22πr2(1−e−2r2/σ2).

This concludes the proof.

**Figure 5:**
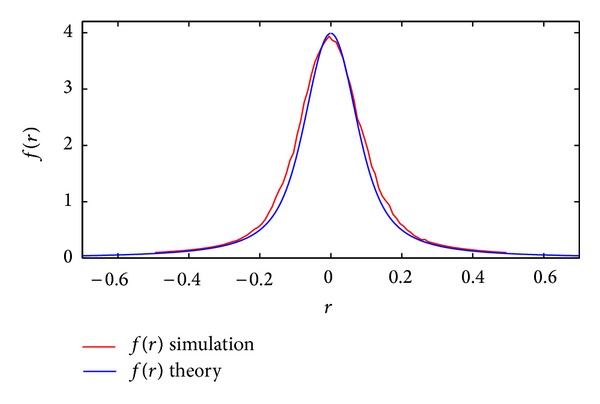
Comparison of theoretically derived (30) and numerically simulated error pdf for *σ*_*e*_ = 0.1.

The numerically simulated overall errors and the shape of the above theoretical distribution are plotted in Figure 5. This figure shows the high accuracy of the above derivation in predicting the shape of errors distribution.

Using Proposition 1, we can model optical flow calculation as (2), where *a*_*i*_ = −*I*_*x*_*i*__/*I*_*y*_*i*__ is the slope,*b*_*i*_ = −*I*_*t*_*i*__/*I*_*y*_*i*__ is the vertical intercept of the *i*th linear equation, and *v*, *u* are the image velocities. Thus, the estimation error of velocity can be calculated as (31).

### 3.2. Definition of a Structure

Definition of a structure is a cornerstone of any data segmentation solution. In classical statistics, the definition of a structure is simpler than computer vision domain as the data is assumed to have only one structure and the structure always has the majority of data [27]. In that context, a structure in data space is defined as a majority of data satisfying |*r* | <*Tσ* in which *r* is a measure of the goodness for a data point, *T* is a constant (normally set between 2 to 3 based on the desired significance level of the Gaussian distribution), and *σ* is an estimate of the scale of measurement noise.

**Figure 6:**
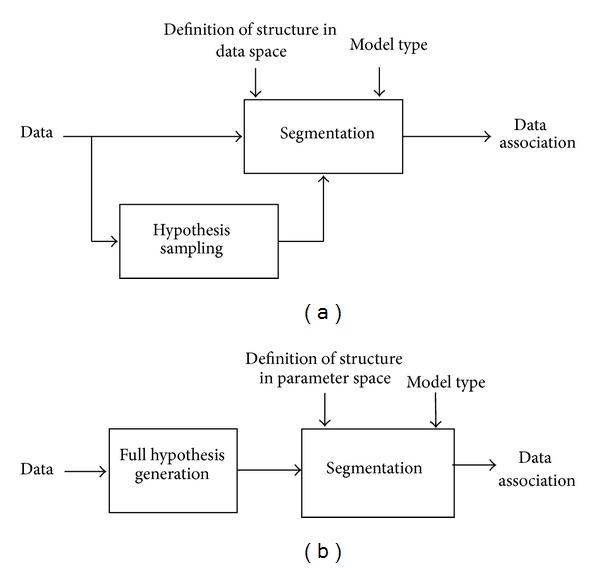
(a) The block diagram of a typical parametric segmentation solution. (b) The block diagram for the proposed analysis of the segmentation problem.

The above represents a circular definition as the *r* in the data space is measured using some attributes of the structure that are being defined. To bypass this issue, as shown in Figure 6(a), the estimation and segmentation are commonly conducted using random or guided sampling [6] and a good (in a statistical sense) sample is used in place of the true model. As it was mentioned earlier, this complicates the analysis of separability as the segmentation results would depend on the method by which the problem is solved. For instance, in the Hough Space segmentation, the definition of a structure and accuracy of segmentation outcomes would depend on the used histogram bandwidth for which the appropriate value is not known a priori.

To address the above issue our analysis, as shown in Figure 6(b), is conducted in the space of all pairwise samples. Our earlier derivation of the probability distribution of those samples enables the development a precise definition for a structure in this space. This definition is the key to solving the separability problem. To develop a unique (noncircular) definition, we use two basic principles that broadly define a cluster in any space.

The first principle guiding our definition is that a structure should be represented by the largest possible set of samples to include all the attributes of the modeled quantity. Also, any putative structure must include more than half of the overall samples to ensure the uniqueness of the definition for a given set of samples.

The second principle guiding our definition is that the probability of a given amount of error for a structure in the sample space should be less than the probability of the measurement noise for the same error value. This means that any data should always be more probable given the true model than any other model (sample).

**Figure 7:**
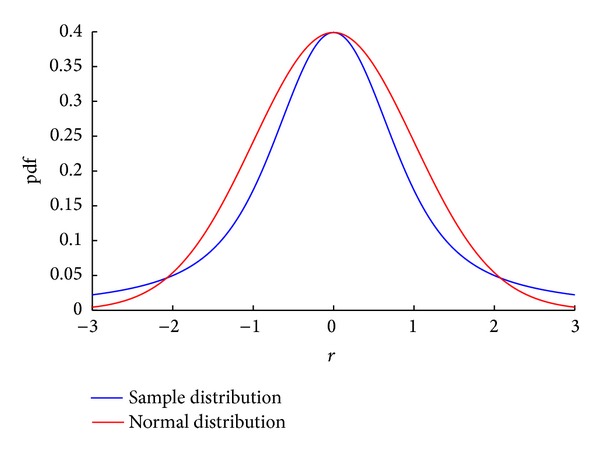
The shape of the theoretically distributed *f*(*r*) and normal distribution for *σ*_*e*_ = 1.

To demonstrate the application of the above principles for defining a structure, we first find the crossover points by equating the derived error pdf (31) with the Gaussian function representing the measurement noise distribution. The result shows that error probabilities of samples up to 2.1 times the measurement noise scale is smaller than the measurement noise probabilities (*f*(*r*) < *f*(*e*)) for all measurement noise scales. This fact, for the case of *σ*_*e*_ = 1, is demonstrated by plotting those functions in Figure 7. Furthermore, the sample size of the above group (with |*r* | <2.1*σ*_*e*_) is significantly larger than half. Exact values of the sample size and its variance up to crossover points are calculated here:

(32) $$\begin{array}{c} \sigma_{r}^{2}=\int_{-2.1\sigma_{e}}^{2.1\sigma_{e}}r^{2}f_{r}\left(r\right)\cdot dr=0.6\sigma_{e}^{2}, \end{array}$$

(33) $$\begin{array}{c} \text{sample}\;\text{size}=\int_{-2.1\sigma_{e}}^{2.1\sigma_{e}}f_{r}\left(r\right)\cdot \;dr\approx 81\%. \end{array}$$

The above calculations show that a very large majority (above 80%) of samples provide a precise representation of the underlying structure. Also, this definition is in-line to its traditional counterpart in the data space as we have (from (32)): $|r|<(2.1/\sqrt{0.6})\sigma_{r}$ or simply |*r* | <2.7*σ*_*r*_.

A structure in sample space is then formally defined as a cluster of at least half the samples where all of its samples satisfy |*r* | <2.1*σ*_*e*_. It is important to note that the above definition includes a large number of samples (more than half of the 2combinations of all samples which is significantly larger than the number of observations) and by definition is robust to influence of outliers [16, 17]. Also, the definition is *independent* of the distribution of samples and only depends on the scale of measurement noise (a design parameter) in the data space.

### 3.3. Segmentation Feasibility

 Having derived the probability density function of error samples and provided a precise definition of a structure in the sample space, we can now examine the feasibility of the segmentation of the system of linear equations representing optical flows in two coherent systems by a simple cluster analysis. The separability of two systems of linear equations with close solutions can now be examined by considering the separability of the sample distributions. We note that two structures, as defined earlier, are separable if the distance between their means is at least 2.1(*σ*_1_ + *σ*_2_), where *σ*_1_ and *σ*_2_ are the standard deviations of their associated measurement noise, respectively.

This statement is simply explained by looking at its contradiction. If we assume that the means of the distributions are already known, it would still not be possible to segment the data cleanly unless the two distributions have no overlap. This implies that the distance between two means has to be at least equal to sum of the extent of those distributions. This presents a sufficient condition for the separability which is able to predict the motion separability using only the amount of relative motion and the scale of noise in the measurement data. It is important to note here that the condition explicitly assumes that there are two distinct motions and in contrast to [18] (and subsequent works that followed this), the flow is not assumed to be varying smoothly.

## 4. Applications

 To examine the usability of proposed theory for motion separability prediction, results of several experiments on standard video sequences for optical flow analysis are discussed. First, a set of controlled experiments using synthetically generated texture were conducted to simulate the separability of similar motions. Both the amount of relative motion and scale of measurement noise were changed in those simulations and the effect of those on the separability of existing motions were analyzed. Then, the application of the proposed theory for prediction of different motions in various video sequences (e.g., from Middlebury) with multiple motions were examined. The result shows that the proposed criterion is capable of predicting the separability of the motion of different objects.

Calculation of image derivatives is an important aspect of optical flow calculation and there are different ways to ensure that image derivatives are not affected by noise and aliasing [28]. Our experiments however showed that although using multiresolution or relatively sophisticated image interpolation techniques (similar to ones used in [9]) improves the appearance of final results, the conclusions remained unchanged. Consequently, and for the sake of simplicity, spatial and temporal derivatives in our experiments were all calculated using convolution with Gaussian filters with the standard deviation of 1 to 2 pixels in all directions.

### 4.1. Simulations

**Figure 8:**
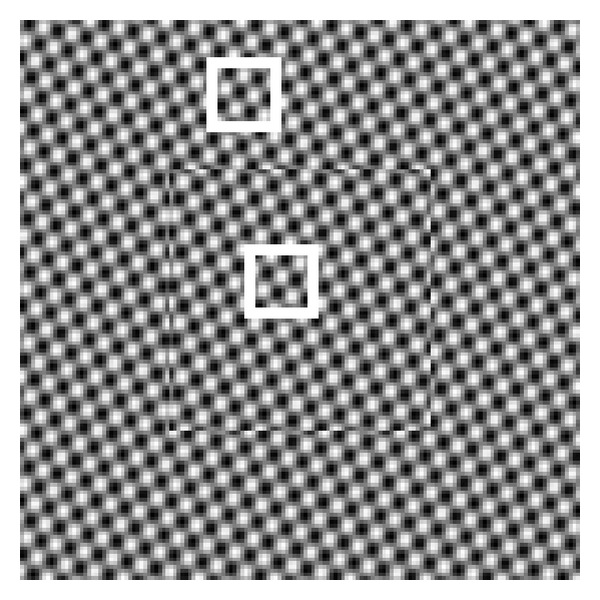
A frame of synthetic image sequence.

The usability of the proposed theory for motion separability predictions is examined here using a sinusoidal synthetic image sequence [5]. The texture of the image sequence is generated by the superposition of two sinusoidal moving plane waves. The central square of the image is stationary, while the surrounding pixels are manipulated to exhibit different constant velocities.  Figure 8 demonstrates a sample frame of the image sequence and highlights (by white rectangles) locations of two patches of size 20 × 20 on both moving and stationary parts. In this simulation, the scale of noise for both patches is the same and therefore the separability condition is (*v*_1_ − *v*_2_) > 2 × 2.1*σ*_*e*_. 

To simulate the effect of noise on the separability predictions, the optical flow constraints for the previously mentioned areas were perturbed by additive Gaussian noise.

**Figure 9:**
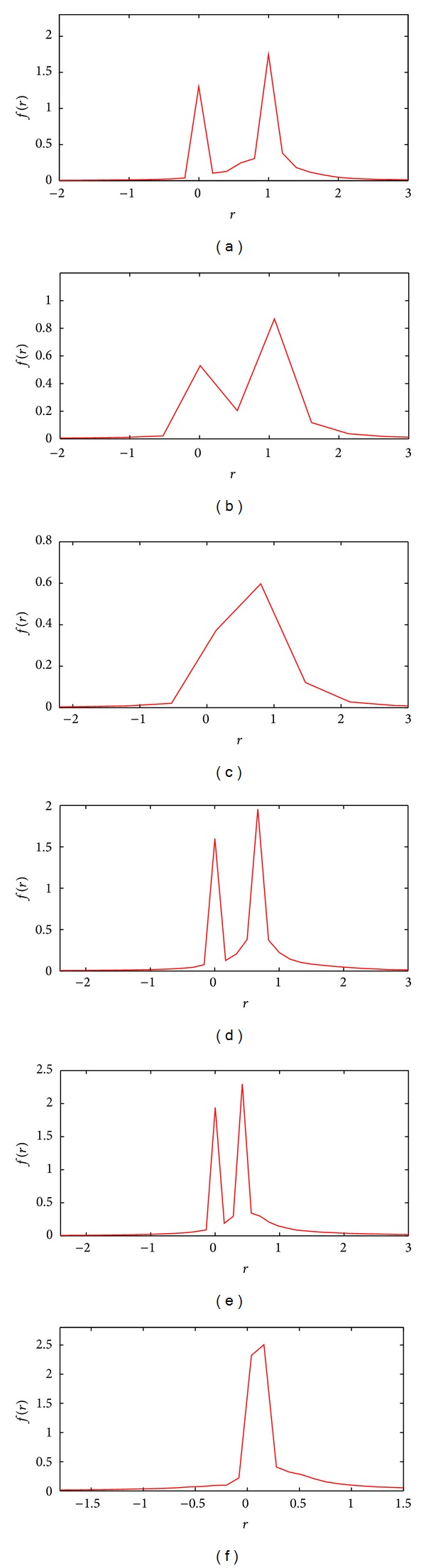
Demonstration of the usefulness of the theoretical separability predictions of two motions having different values of relative motions and noise scales. The distributions of residuals for the combined patches shown in Figure 8 for *v*_1_ − *v*_2_ = 1 and different noise scales including (a) *σ*_*e*_ = 0.14 (separable), (b) *σ*_*e*_ = 0.24 (critical), and (c) *σ*_*e*_ = 0.45 (inseparable), as well as different relative velocities for *σ*_*e*_ = 0.1 including (d) *v*_1_ − *v*_2_ = 0.7 (separable), (e) *v*_1_ − *v*_2_ = 0.42 (critical), and (f) *v*_1_ − *v*_2_ = 0.2 (inseparable).

In the first simulation, the normalized histogram of all samples for both patches containing two motions for different scales of added noise is shown in Figures 9(a)–9(c). Using the above separability condition (*v*_1_ − *v*_2_) > 4.2*σ*_*e*_, we would predict that when *σ*_*e*_ is less than 0.24, the above motions are separable. To show the validity of this prediction, the normalized histograms for values less, at, and above 0.24 are shown. Those figures show that for *σ*_*e*_ < 0.24, two motions are clearly separable.

In the second simulation, the result of changing the relative velocity for a given amount of noise (*σ*_*e*_ = 0.1) is examined. The normalized histogram for velocities less than, at, and greater than the predicted values are also shown in Figures 9(d)–9(f). These figures again show that as long as (*v*_1_ − *v*_2_) > 0.42 then the two motions are separable.

### 4.2. Real Image Experiments

**Figure 10:**
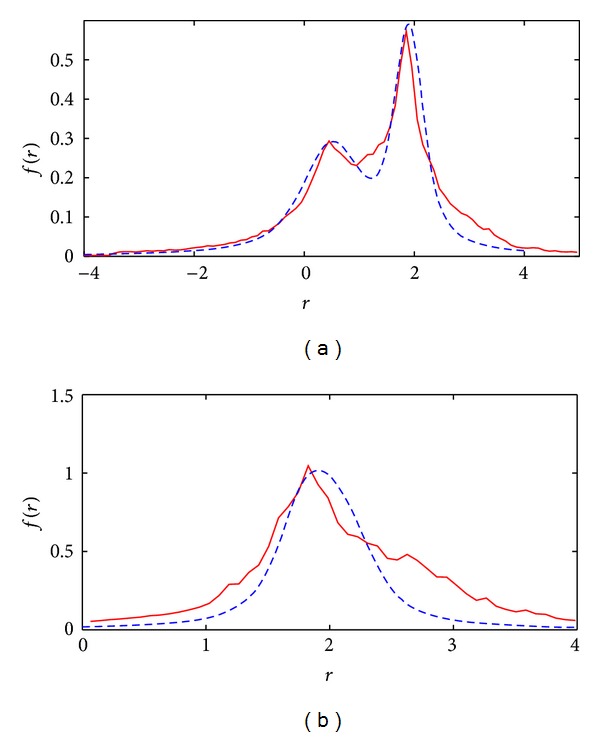
(a) and (b) The theoretical (dashed) distributions of residuals for Figure 1.

As for real data usability, at the beginning, we raised the question of how to predict the least amount of required relative motion between different objects in the Marbled Block sequence (shown in Figure 1) that would make those separable. The proposed theory is now able to predict the separability of different objects using their motion information. Considering that the scale of noise for calculation of local optical flow in this sequence is measured to be around 0.35 pixels/frame, the proposed separability condition, (*v*_1_ − *v*_2_) > 2.1(*σ*_1_ + *σ*_2_), predicts that if the relative motion is greater than 2.1 × 2 × 0.35 = 1.5, those motions are separable. In Figure 1 the relative motion between the two highlighted areas that are located on two different columns is around *v*_1_ − *v*_2_ = 2.1 − 0.5 = 1.6 and therefore we expect those to be separable. Both the theoretical and actual distributions of the flow samples for the combined data are shown in Figure 10(a). In contrast, the maximum relative motion of the areas on one column is around *v*_1_ − *v*_2_ = 2.1 − 1.8 = 0.3 and since it is less than the above separability threshold, those motions are expected to be inseparable. Again, the theoretical and actual distributions of the flow samples for the combined data of the two highlighted areas on a single column are shown in Figure 10(b).

**Figure 11:**
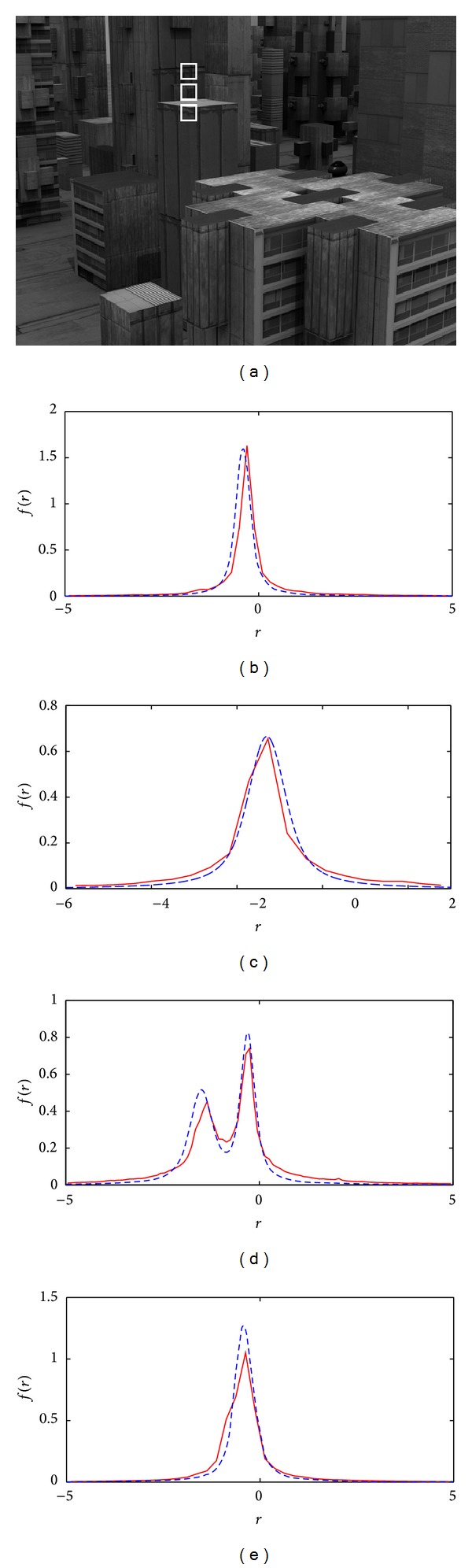
(a) A sample frame of the Urban2 image sequence. (b) and (c) Theoretical (dashed) and measured (solid) pdf of *r* for two different patches on two different objects highlighted by white rectangles on the sample frame. (d) and (e) Joint pdf of *r* values for two patches: (d) of two different objects and (e) of the same object.

**Figure 12:**
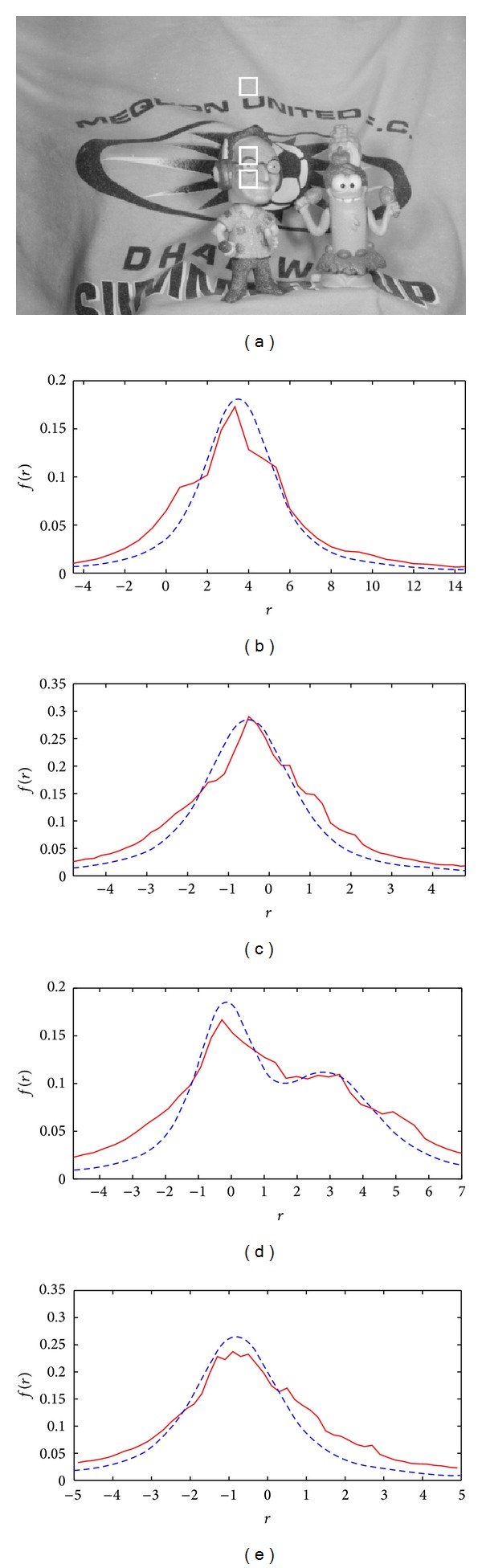
(a) A sample frame of the Mequon image sequence. (b) and (c) Theoretical (dashed) and measured (solid) pdf of *r* for two different patches on two different objects highlighted by white rectangles on the sample frame. (d) and (e) Joint pdf of *r* values for two patches: (d) of two different objects and (e) of the same object.

**Figure 13:**
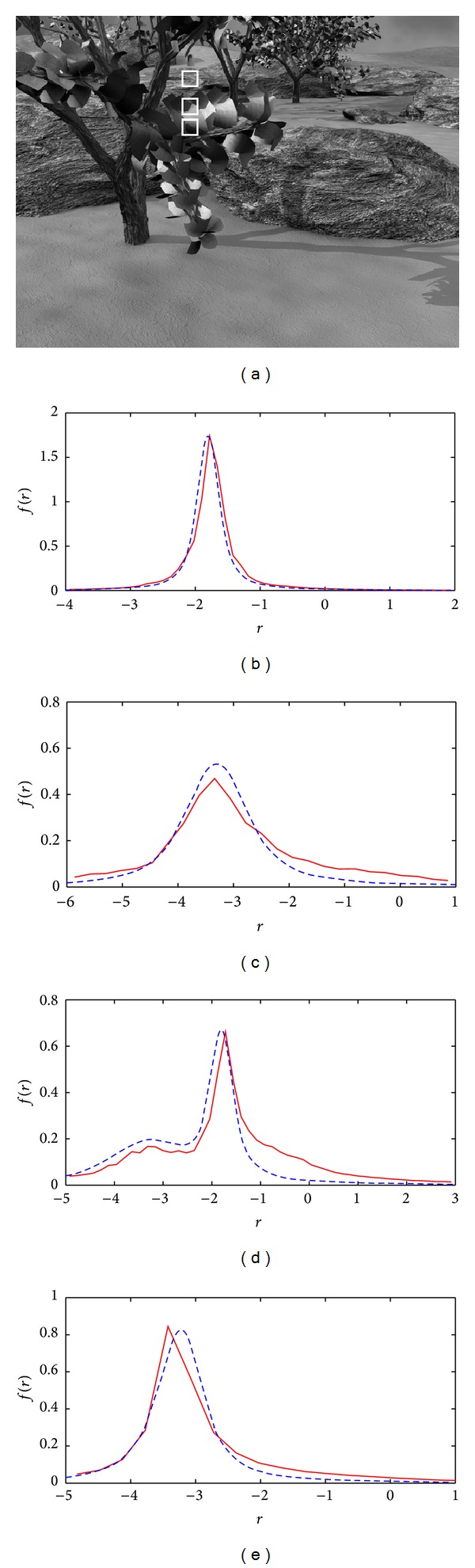
(a) A sample frame of the Grove2 image sequence. (b) and (c) Theoretical (dashed) and measured (solid) pdf of *r* for two different patches on two different objects highlighted by white rectangles on the sample frame. (d) and (e) Joint pdf of *r* values for two patches: (d) of two different objects and (e) of the same object.

**Figure 14:**
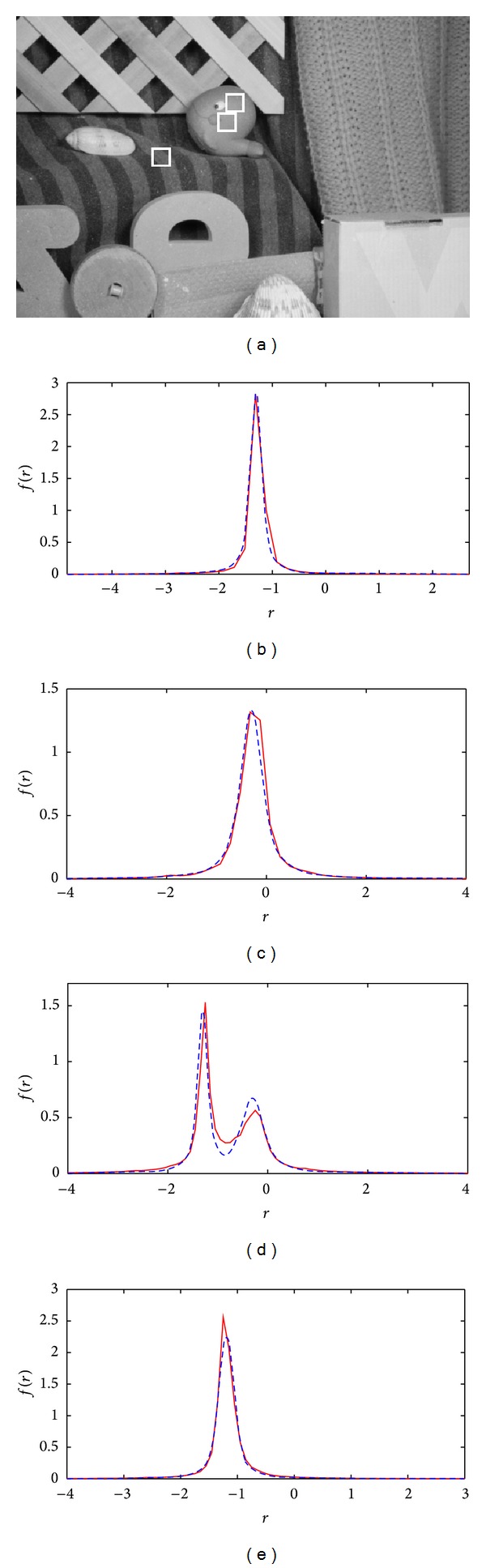
(a) A sample frame of the RubberWhale image sequence. (b) and (c) Theoretical (dashed) and measured (solid) pdf of *r* for two different patches on two different objects highlighted by white rectangles on the sample frame. (d) and (e) Joint pdf of *r* values for two patches: (d) of two different objects and (e) of the same object.

To demonstrate the application of the proposed separability prediction criterion, the separability of different moving objects in a number of image sequences that are commonly used for motion analysis including four from the Middlebury [21] (called Urban2, Mequon, Grove2 and RubberWhale) benchmarks was examined. In all of those sequences, three patches on two different moving objects were chosen and those are highlighted in part (a) of Figures 11, 12,13, and 14. Patches that are on one object and have very similar motions are not expected to be separable. However, the other patch which is on a different object and has sufficiently different motion is expected to be separable from either of those patches. For each image sequence, both the measured and analytical error probability distributions for two different patches of two different objects (shown in parts (b) and (c) of Figures 11–14) as well as distributions of the joint patches of the same and different objects (shown in parts (d) and (e) of Figures 11–14) were plotted. Those plots show that the analytically derived probability for different patches is close to their real values. The numerical results of the above experiments in terms of relative motion between different patches, average scale of noise for the image sequence, and the separability verdict of pairs of patches are provided in Table 1. 

The validity of the above predictions provides evidence that the proposed theory is able to correctly predict the separability of motion for real world applications.

## 5. Conclusion

 A new theoretical framework to predict the feasibility of optical flow segmentation is presented. The framework enables the theoretical derivation of the optical flow estimation error probability density function as well as a precise definition for a visual structure based on its motion. The combination of these two elements is used to develop a segmentation feasibility criterion that can predict the separability of multiple motions. Applications of the theoretical results for the prediction of the separability of multiple motions were examined using both synthetic and real image sequences. The result illustrates that the proposed criterion is able to correctly predict the separability in those cases.

## Figures and Tables

**Table 1:** Numerical results of real image experiments and separability predictions between different patches.

Sequence	*v*_1_ − *v*_2_	*v*_1_ − *v*_3_	*σ*_*e*_	1⇌2	1⇌3
RubberWhale	0.8	0.2	0.15	*✓*	×
Mequon	3.5	0.4	0.8	*✓*	×
Urban2	1.1	0.1	0.41	*✓*	×
Grove2	1.5	0.2	0.26	*✓*	×

(*✓* indicates separability, × otherwise).
